# Supplementary figures and images for: Deficiency of Polη in Saccharomyces cerevisiae reveals the impact of transcription on damage-induced cohesion
Source: PLoS Genet. 2021 Sep 9;17(9):e1009763. doi: 10.1371/journal.pgen.1009763 (PMC8454932; doi:10.1371/journal.pgen.1009763)

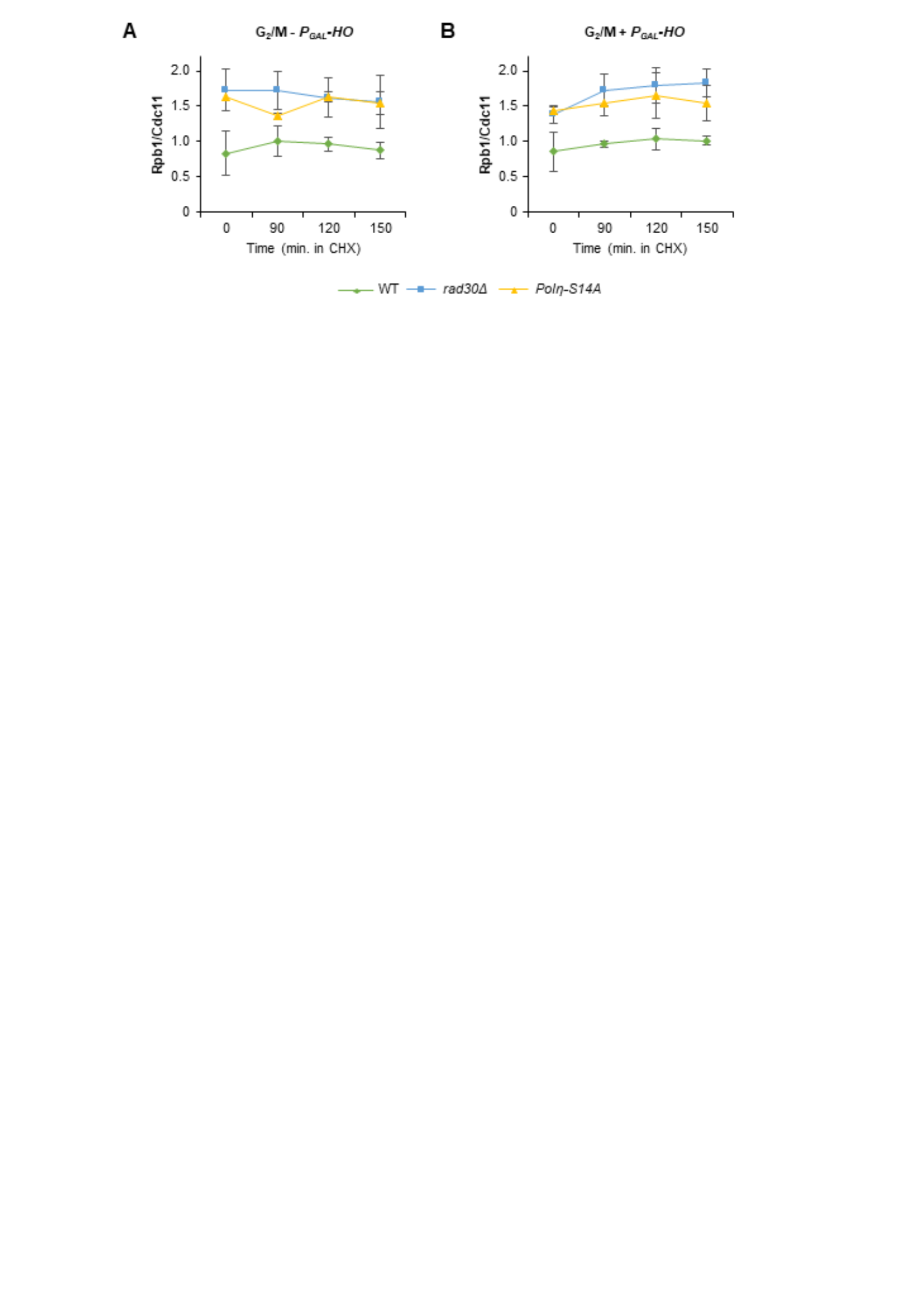

Supplement: S1 Fig — (A-B) Relative amounts of Rpb1 after addition of water (A, control) or galactose (B) to induce PGAL-HO DSB induction for one-hour, followed by cycloheximide (CHX) chase up to 150 minutes. Western blots from two independent experiments were quantified to compare Rpb1 levels (relative to Cdc11) between the indicated strains. (TIFF) [file pgen.1009763.s001.tiff]

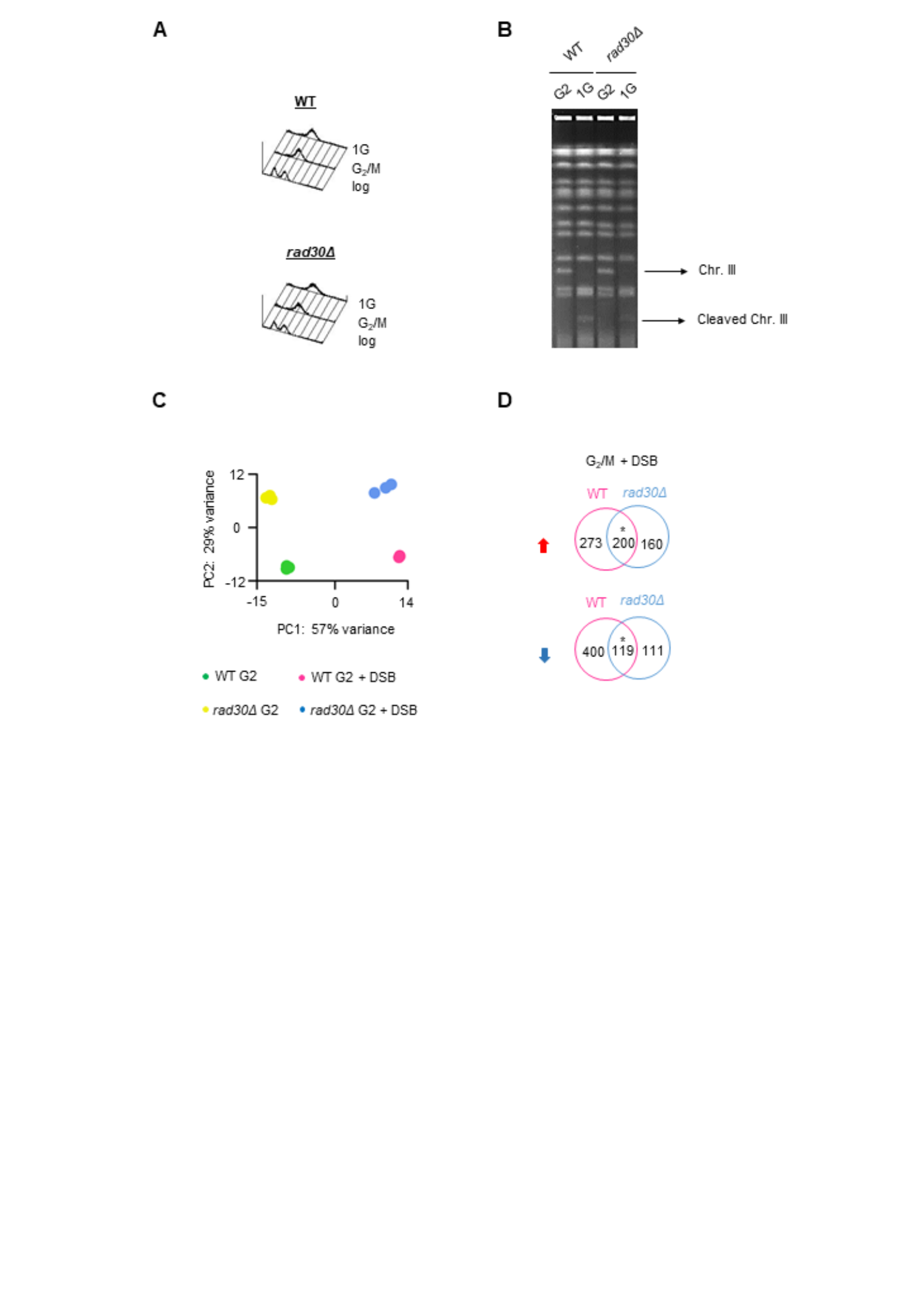

Supplement: S2 Fig — (A) FACS analysis to confirm benomyl-induced G2/M arrest. 1G, 1-hour GAL-induction (PGAL-HO). (B) PFGE analysis to monitor DSB induction on chromosome III. G2, G2/M arrest; 1G as in (A). (C) PCA demonstrating distribution of independent data sets between groups and clustering of data sets within groups. (D) Venn diagrams showing overlaps of differentially expressed genes in WT and rad30Δ cells after DSBs, based on RNA-seq. The red and blue arrows indicate up- and down-regulated genes respectively. Statistical significance of the overlapping genes was evaluated as described in Materials and Methods, with * p < 0.001. (TIFF) [file pgen.1009763.s002.tiff]

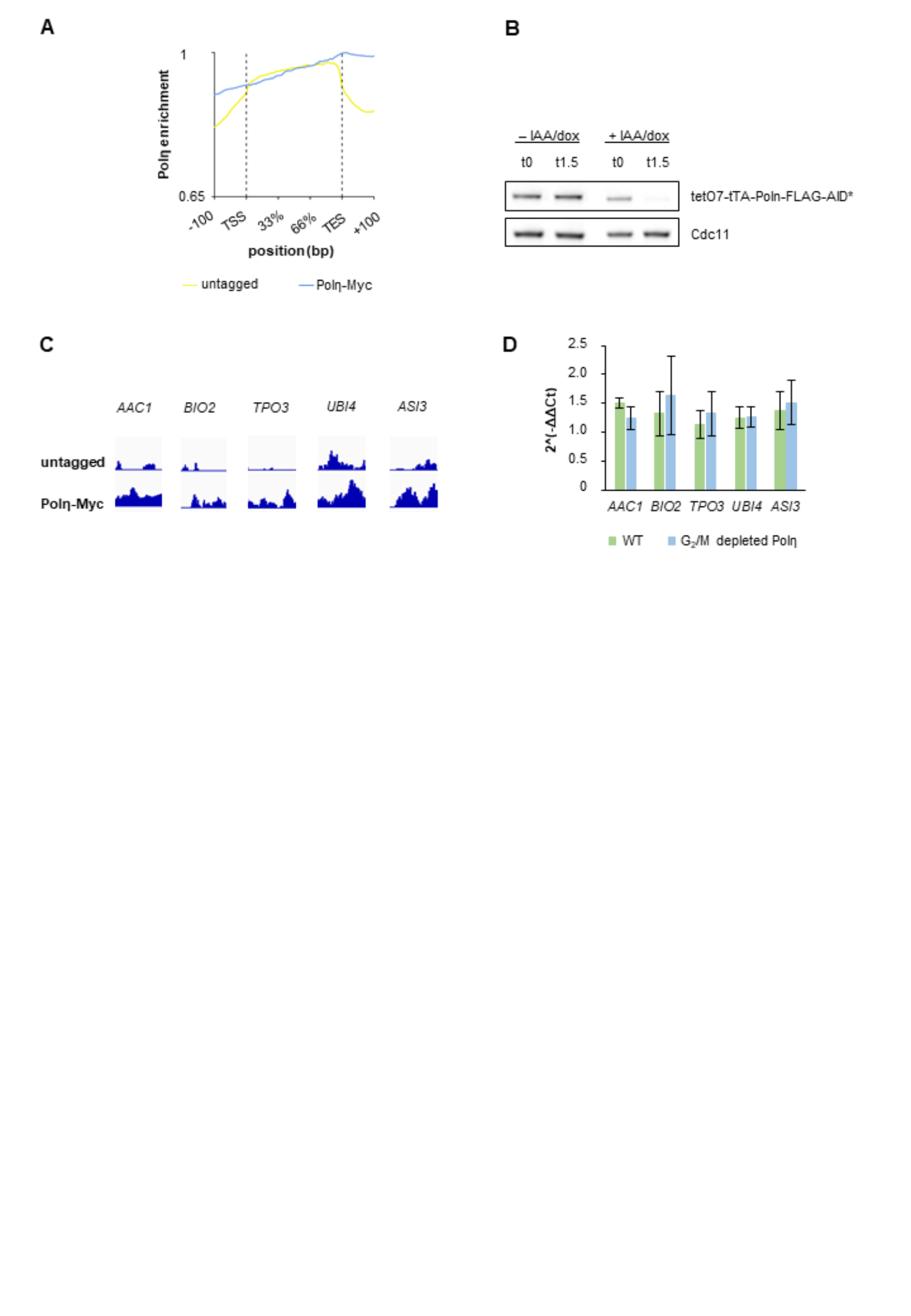

Supplement: S3 Fig — (A) Metagenome plot showing distribution of Polη, with 100 bp flanking regions upstream and downstream of the gene bodies during G2/M phase. The samples were first normalized to their respective input and then the values were scaled to the maximum value of the plot. (B) Western blot to check depletion of Polη in G2/M arrested cells. Final concentrations of auxin and doxycycline were 6 mM and 20 μg/ml respectively. IAA, auxin; dox, doxycycline; t0, the 0-time point after addition of IAA/dox; t1.5, 90 minutes after treatment. The drug solvents (50% ethanol and water) were added in the ‘-IAA/dox’ mock control. The western blot image, including the protein marker, was cropped to show selected samples. Cdc11 was used as loading control. (C) Representative Integrative Genomics Viewer (IGV) tracks showing the differences in distribution of Polη at selected promoters. The samples were normalized to their respective input and library size. (D) Expression of selected genes with or without depletion of Polη during G2/M, measured by RT-qPCR. Calculations were the same as described in the legend of Fig 3F. Error bars indicate the mean ± STDEV of three independent experiments. (TIFF) [file pgen.1009763.s003.tiff]

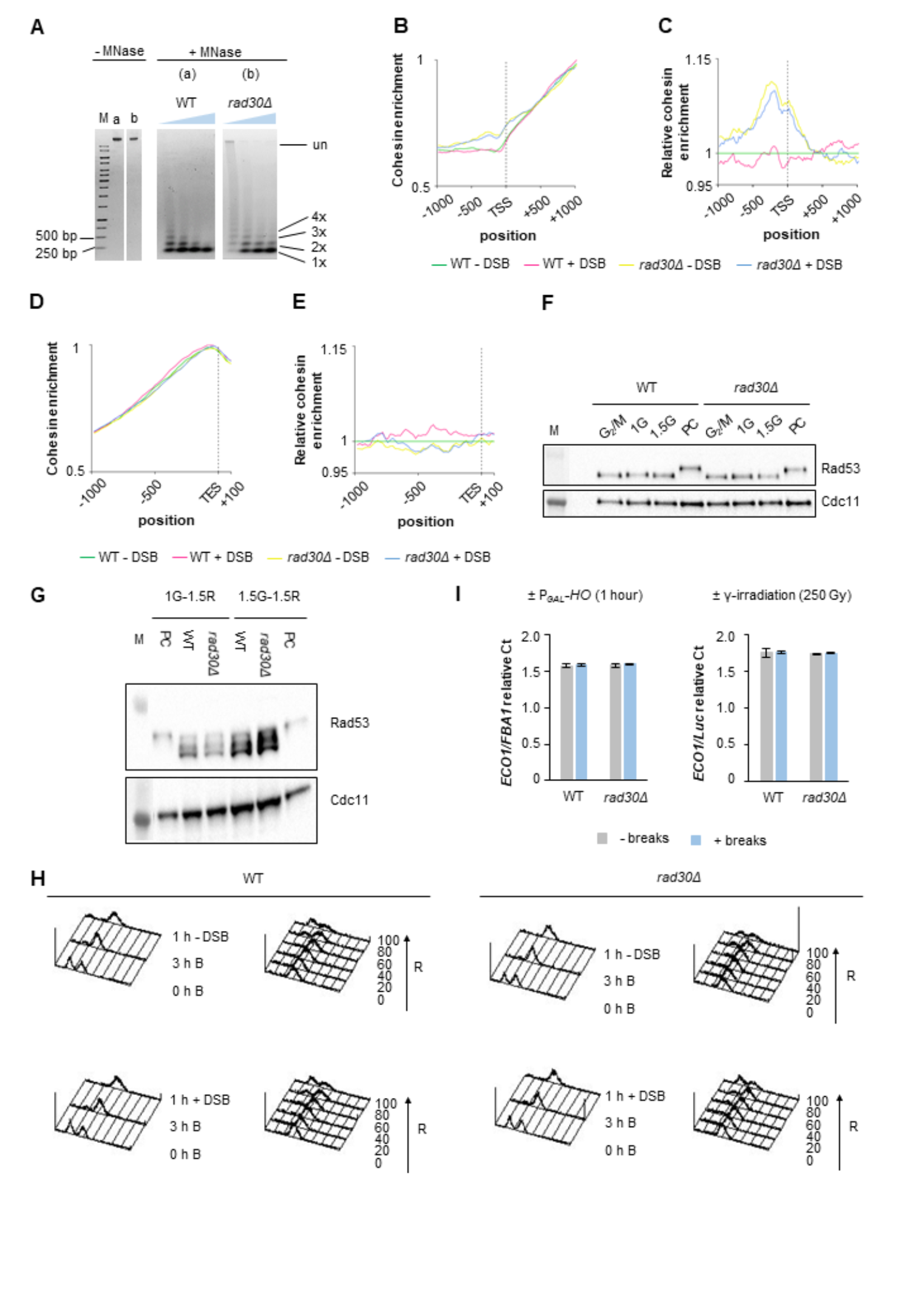

Supplement: S4 Fig — (A) Monitoring nucleosome occupancy based on sensitivity of cells to MNase digestions. The concentrations of MNase were 0, 0.0125, 0.025, 0.05, 0.1 U/ml (final). One representative gel electrophoresis from at least two independent assays performed is shown. The gel images were cropped to show selected samples. M, DNA ladder; Un, undigested; 1x, monomer; 2x, dimer; 3x, trimer; 4x, tetramer. (B) Metagenome plot showing cohesin enrichment ± 1000 bp from the transcription start site (TSS) in WT and rad30Δ cells ± DSB induction in G2/M phase. The samples were first normalized to their respective input and then the values were scaled to the maximum value of the plot. (C) The data from (B) plotted relative to the WT-DSB sample. After normalizing to the input, all samples were also normalized to WT-DSB sample to visualize the changes between the WT and rad30Δ cells. (D) Metagenome plot showing cohesin distribution 1000 bp downstream and 100 bp upstream from the transcription end site (TES) in WT and rad30Δ cells ± DSB induction in G2/M phase. Plotted as in (B). (E) As in (C), except plotting cohesin distribution around the TES according to (D). (F) Monitoring activation of the DNA damage checkpoint (phosphorylation of Rad53) after DSB induction with western blot. Galactose was added into the G2/M arrested cell cultures to induce PGAL-HO break induction for 1- or 1.5-hour, denoted as 1G or 1.5G. Sample collected from G2/M arrested WT cells, treated with phleomycin (final 15 μg/ml) for 1.5 hours was included as positive control (PC). Cdc11 was used as loading control. M, protein marker. (G) Monitoring activation of DNA damage checkpoint during DSB recovery. DSBs were induced for 1- or 1.5-hour, as in (F). The cells were then allowed to recover in YEP media supplemented with glucose and benomyl for another 1.5 hour (1.5 R) at 35°C, to mimic the damage-induced cohesion assay. 1G, 1.5G, PC, M as in (F). Cdc11 was used as loading control. (H) FACS analyses of cell cycle progressi [file pgen.1009763.s004.tiff]

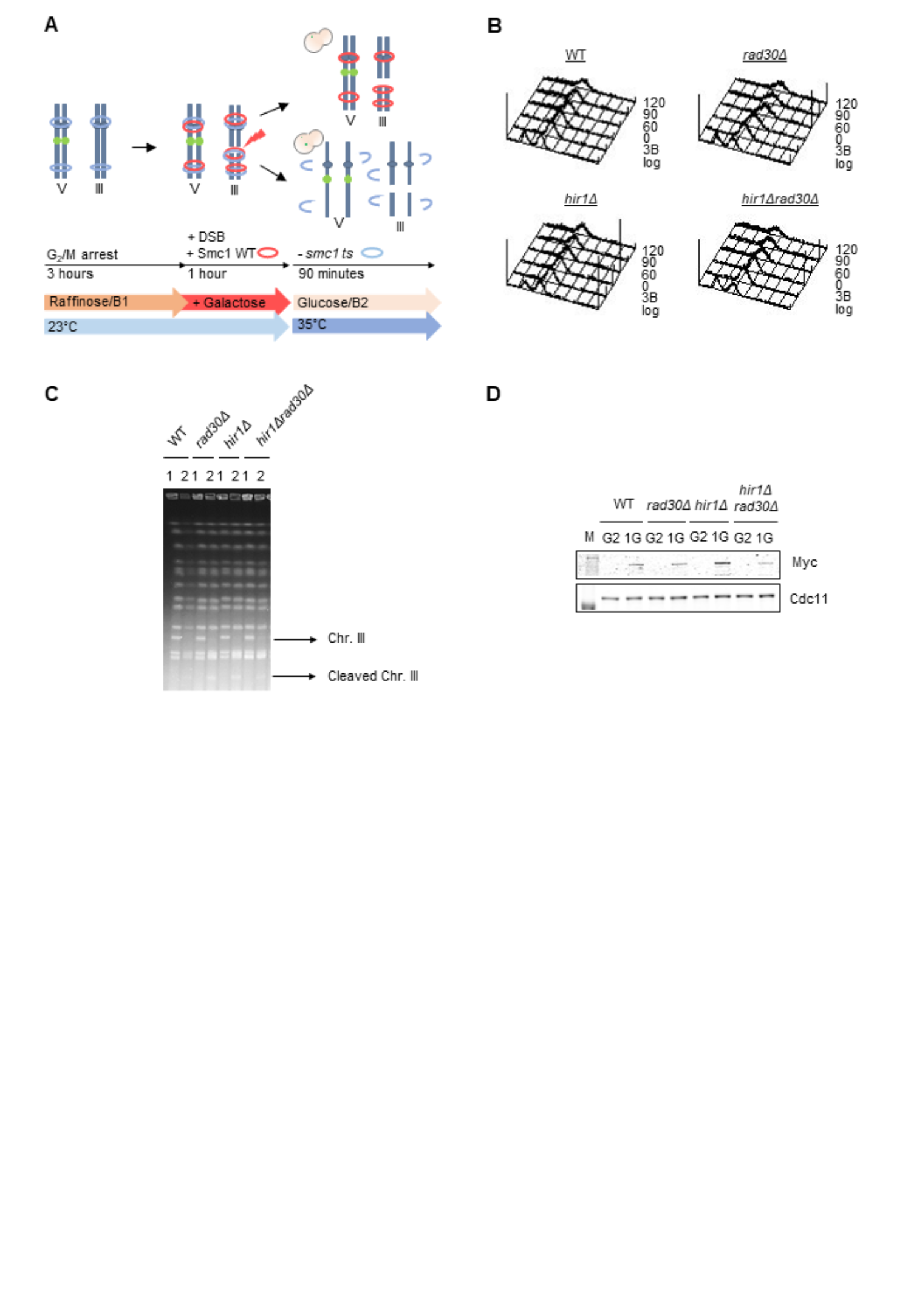

Supplement: S5 Fig — (A) Damage-induced cohesion assay performed with GAL induced DSBs on chromosome III (PGAL-HO). Strains harboring the temperature sensitive smc1-259 allele are arrested in G2/M by addition of benomyl (‘B’). Galactose is then added for expression of ectopic PGAL-SMC1-MYC (Smc1 WT) and induction of DSBs, for 1-hour. The temperature is then raised to 35°C, restrictive to the smc1-259 allele, for disruption of S-phase cohesion (blue rings). The Tet-O/TetR-GFP system (green dots) is used to monitor damage-induced cohesion (red rings) on chr. V. Chr., chromosome; III, three; V, five. B1 and 2 indicate replacement of media with freshly prepared benomyl. (B) FACS analysis to confirm G2/M arrest during the time course of a typical damage-induced cohesion assay. 3B, 3-hour benomyl arrest. (C) PFGE analysis to detect DSB induction on chromosome III. 1, G2/M arrest; 2, 1-hour GAL-induction (PGAL-HO and PGAL-SMC1-MYC). (D) Western blot to check expression of the GAL promoter driven ectopic Smc1-Myc protein. G2, G2/M arrest; 1G, 1-hour GAL-induction as in (C). Cdc11 was used as loading control. M, protein marker. (TIFF) [file pgen.1009763.s005.tiff]

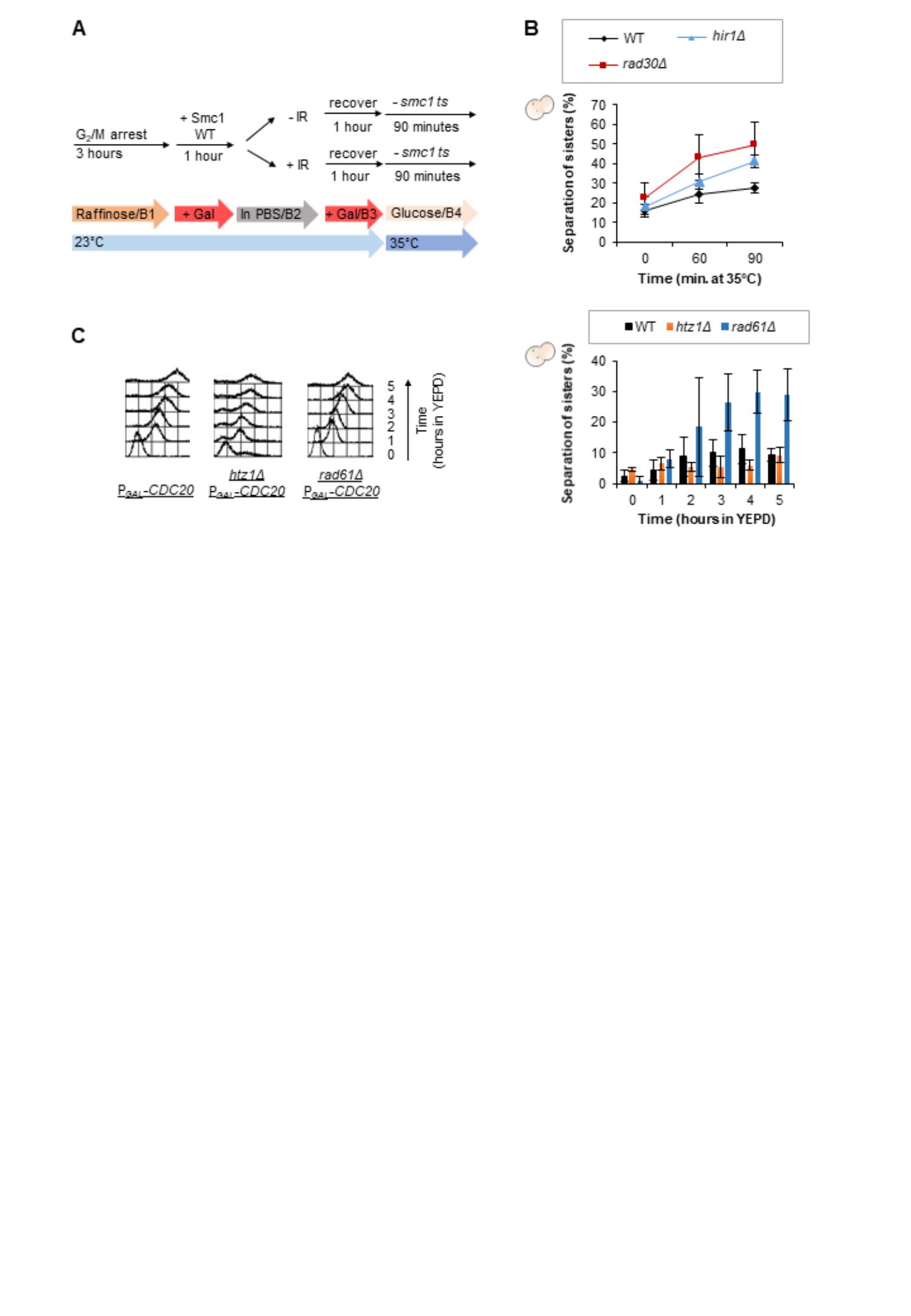

Supplement: S6 Fig — (A) Damage-induced cohesion assay performed with γ-irradiation. Formation of damage-induced cohesion is monitored on chr. V with the same Tet-O/TetR-GFP system, as in S5A Fig, with slight differences in the experimental procedure. Strains with smc1-259 background are arrested in G2/M by addition of benomyl (‘B’), expression of ectopic PGAL-SMC1-MYC (Smc1 WT) is then induced by addition of galactose. The cells are subsequently pelleted, resuspended in 1X PBS supplemented with benomyl. The resuspension is split in one half for irradiation, and half as non-irradiated control. After irradiation, both ± irradiated cells are recovering in YEP media supplemented with galactose and benomyl. Subsequently, the media is changed to YEP containing glucose and benomyl, and the temperature raised to 35°C, to monitor formation of damage-induced cohesion. (B) Damage-induced cohesion assay of the hir1Δ mutant in response to γ-irradiation, performed as depicted in (A). Means ± STDEV from two independent experiments are shown. For each experiment, two-hundred cells were counted for each time point. (C) Sister chromatid cohesion maintenance of the htz1Δ mutant under prolonged G2/M arrest. The cells were initially synchronized in G1 by α-factor in YEP media containing galactose. Expression of PGAL-CDC20 was then shut off by switching the carbon source to glucose (YEPD), which resulted in the subsequent prolonged G2/M arrest as monitored by FACS (left panel). Sister chromatid separation was monitored at the URA3 locus on Chr. V by the TetO/TetR-GFP system. Means ± STDEV from three independent experiments are shown (right panel). A rad61Δ mutant with known high sister separation under prolonged G2/M arrest was included as control. Parts of the results from the same experiments were previously published [28]. Chr., chromosome. (TIFF) [file pgen.1009763.s006.tiff]

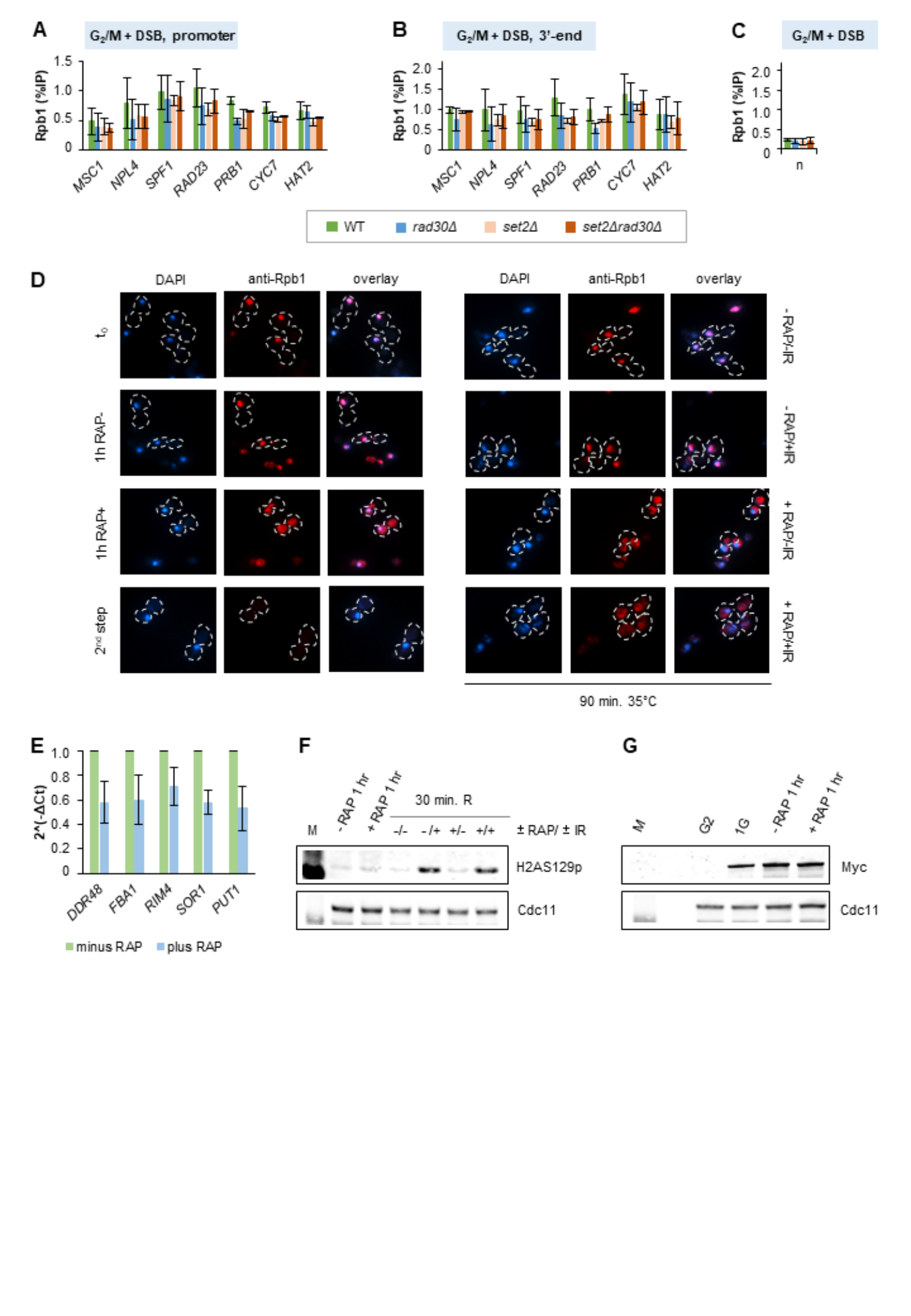

Supplement: S7 Fig — (A-C) ChIP-qPCR analyses to determine chromatin association of Rpb1 at promoters and 3’-ends of selected genes, in G2/M arrested cells after DSB induction. The same genes as in Fig 7C–7E were analyzed. Error bars indicate the mean ± STDEV of three independent experiments. n, low-binding control (n2 in Fig 1B). (D) Representative in situ immunofluorescence images for samples collected from the damage-induced cohesion assays in Fig 7F. The cells were stained with anti-Rpb1 and then counterstained with DAPI. t0, the time point before splitting the culture for addition of rapamycin (RAP); 1h RAP, 1-hour after ± rapamycin; 2nd step, the secondary antibody alone as control. (E) Fold reduction of selected genes after 1-hour rapamycin treatment, measured by RT-qPCR. The 2-ΔCt values of untreated samples were set as 1. (F) Western blot to monitor early DNA damage response, as indicated by H2AS129-phosphorylation. RAP, rapamycin; R, recovery; IR, γ-irradiation (250 Gy); M, protein marker. Cdc11 was used as loading control. (G) Western blot to check expression of the ectopic Smc1-Myc, driven by the GAL promoter. G2, G2/M arrest; 1G, 1-hour GAL-induction. RAP, M, Cdc11 as in (F). (TIFF) [file pgen.1009763.s007.tiff]

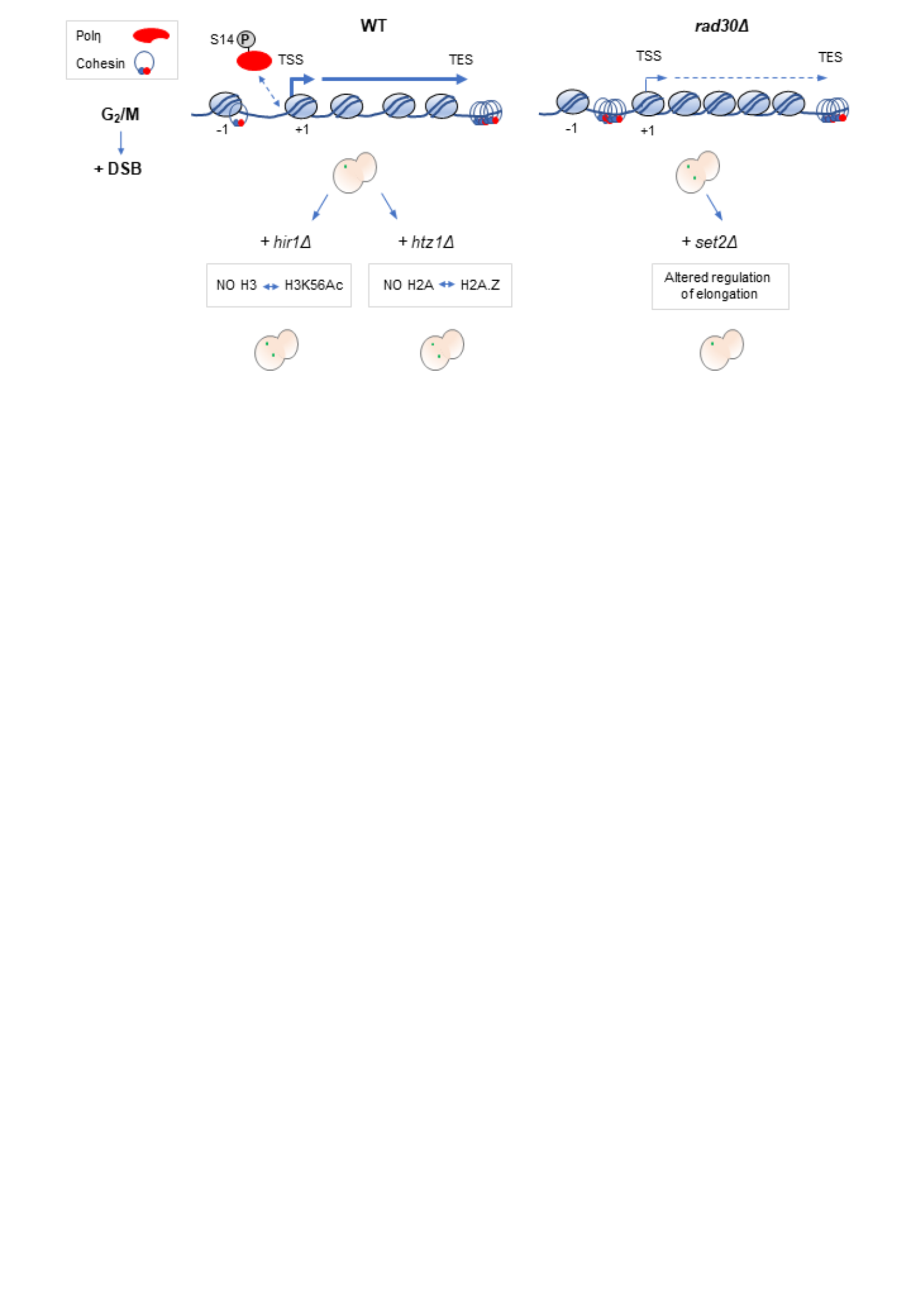

Supplement: S8 Fig — In G2/M arrested WT cells, genes belonging to the positive transcription regulation and chromatin assembly pathways are enriched compared to rad30Δ cells. Reduced chromatin assembly in rad30Δ cells results in less dynamic chromatin, indicated by additional nucleosomes. Deregulated transcription and sensitivity to elongation inhibitors in rad30Δ cells are indicated by thin arrows over the TSS and ORF. Histone exchange between H3 and the post-translationally modified H3 (H3K56Ac) at promoter regions is reduced in the hir1Δ mutant, while histone exchange of H2A.Z for H2A predominantly at the +1 nucleosome is prevented in the htz1Δ mutant, hampering transcriptional regulation. Both mutants were deficient in damage-induced cohesion. In contrast, deletion of SET2 compensated for reduced transcriptional capacity of the rad30Δ mutant, and suppressed the lack of damage-induced cohesion in rad30Δ cells. Taken together, histone exchange during transcription may facilitate formation of damage-induced cohesion. Transcriptional regulation is perturbed in rad30Δ cells, and this appeared to have a consequence on generation of damage-induced cohesion. Cells with a single green dot indicates established damage-induced cohesion while cells with two dots indicates lack of damage-induced cohesion. Since Polη may play an indirect role in transcription, recruitment of Polη to the promoter region is indicated with a dashed double ended arrow. ORF, open reading frame. (TIFF) [file pgen.1009763.s008.tiff]
