# Supplementary material for: Deficiency of Polη in Saccharomyces cerevisiae reveals the impact of transcription on damage-induced cohesion
Source: PLoS Genet. 2021 Sep 9;17(9):e1009763. doi: 10.1371/journal.pgen.1009763 (PMC8454932; doi:10.1371/journal.pgen.1009763)
Supplement: S1 Table — (DOCX) [file pgen.1009763.s014.docx]

| **S1 Table. Strains used in this study** | |
| --- | --- |
| **Strain (LS)** | **Genotype** |
| 50/408 | *MATa ade3::GALHO ade2-1 trp1-1 can1-100 leu2-3,112 his3-11,15 ura3-1 GAL psi+ RAD5* |
| 421 | *MATα rad30::KAN smc1-259 ade3::GALHO leu2-3,112::PGAL-SMC1-MYC-LEU2 his3-11,15::HIS3-tetR-GFP ura3-1::tetOx112-URA3 ade2-1 trp1-1 can1-100 GAL psi+ RAD5* |
| 468 | *MATα rad30(S14A)::NAT smc1-259 ade3::GALHO leu2-3,112::PGAL-SMC1-MYC-LEU2 his3-11,15::HIS3-tetR-GFP ura3-1::tetOx112-URA3 ade2-1 trp1-1 can1-100 GAL psi+ RAD5* |
| 496 | *MATa ade::GALHO RAD30-3His-13MYC::KAN ade2-1 trp1-1 can1-100 leu2-3,112 his3-11,15 ura3-1 GAL psi+ RAD5* |
| 514 | *MATa rad30::KAN ade3::GALHO ade2-1 trp1-1 can1-100 leu2-3,112 his3-11,15 ura3-1 GAL psi+ RAD5* |
| 655 | *MATa smc1-259 ade3::GALHO leu2-3,112::PGAL-SMC1-MYC-LEU2 his3-11,15::HIS3-tetR-GFP ura3-1::tetOx112-URA3 ade2-1 trp1-1 can1-100 GAL psi+ RAD5* |
| 656/1098 | MAT*α smc1-259 ade3::GALHO leu2-3,112::PGAL-SMC1-MYC-LEU2 his3-11,15::HIS3-tetR-GFP ura3-1::tetOx112-URA3 ade2-1 trp1-1 can1-100 GAL psi+ RAD5* |
| 657 | *MATa smc1-259 leu2-3,112::PGAL-SMC1-MYC-LEU2 his3-11,15::HIS3-tetR-GFP ura3-1::tetOx112-URA3 ade2-1 trp1-1 can1-100 GAL psi+ RAD5* |
| 666 | *MATa smc1-259 leu2-3,112::PGAL-SMC1-MYC-LEU2 trp1-1::PMET3-CDC20-TRP1 his3-11,15::HIS3-tetR-GFP ura3-1::tetOx112-URA3 ade2-1 can1-100 GAL psi+ RAD5* |
| 699 | *MATa htz1::HPH smc1-259 leu2-3,112::PGAL-SMC1-MYC-LEU2 trp1-1::PMET3-CDC20-TRP1 his3-11,15::HIS3-tetR-GFP ura3-1::tetOx112-URA3 ade2-1 can1-100 GAL psi+ RAD5* |
| 847 | *MATa trp1-1::PGAL-CDC20-TRP1(K. lactis) his3-11,15::HIS3-tetR-GFP ura3-1::tetOx112-URA3 ade2-1 can1-100 leu2-3,112 GAL psi+ RAD5* |
| 848 | *MATa htz1::HPH trp1-1::PGAL-CDC20-TRP1(K. lactis) his3-11,15::HIS3-tetR-GFP ura3-1::tetOx112-URA3 ade2-1 can1-100 leu2-3,112 GAL psi+ RAD5* |
| 910 | *MATa hir1::NAT smc1-259 ade3::GALHO leu2-3,112::PGAL-SMC1-MYC-LEU2 his3-11,15::HIS3-tetR-GFP ura3-1::tetOx112-URA3 ade2-1 trp1-1 can1-100 GAL psi+ RAD5* |
| 914 | *MATα hir1::NAT rad30::KAN smc1-259 ade3::GALHO leu2-3,112::PGAL-SMC1-MYC-LEU2 his3-11,15::HIS3-tetR-GFP ura3-1::tetOx112-URA3 ade2-1 trp1-1 can1-100 GAL psi+ RAD5* |
| 928 | *MATa rad61::KAN trp1-1::PGAL-CDC20-TRP1(K. lactis) his3-11,15::HIS3-tetR-GFP ura3-1::tetOx112-URA3 ade2-1 can1-100 leu2-3,112 GAL psi+ RAD5* |
| 963 | *MATa hir1::HPH smc1-259 leu2-3,112::PGAL-SMC1-MYC-LEU2 his3-11,15::HIS3-tetR-GFP ura3-1::tetOx112-URA3 ade2-1 trp1-1 can1-100 GAL psi+ RAD5* |
| 1002 | *MATα rad30::KAN smc1-259 leu2-3,112::PGAL-SMC1-MYC-LEU2 his3-11,15::HIS3-tetR-GFP ura3-1::tetOx112-URA3 ade2-1 trp1-1 can1-100 GAL psi+ RAD5* |
| 1049 | *MATα smc1-259 ade3::GALHO tetO7-tTA:HIS-RAD30-6XFLAG-AID(71-114)::HPH ura3-1::ADH1-OsTIR1-9MYC::URA3 leu2-3, 112::tetR'-SSN6::LEU2 10Kb-CEN4::LacO-NAT his3-11, 15::GFP-LacI-HIS3 trp1-1::PGAL-SMC1-MYC-TRP1 can1-100 RAD5* |
| 1054 | *MATa hhf1-hht1::HPH smc1-259 ade3::GALHO leu2-3,112::PGAL-SMC1-MYC-LEU2 his3-11,15::HIS3-tetR-GFP ura3-1::tetOx112-URA3 ade2-1 trp1-1 can1-100 GAL psi+ RAD5* |
| 1055 | *MATa hht2-hhf2::HPH smc1-259 ade3::GALHO leu2-3,112::PGAL-SMC1-MYC-LEU2 his3-11,15::HIS3-tetR-GFP ura3-1::tetOx112-URA3 ade2-1 trp1-1 can1-100 GAL psi+ RAD5* |
| 1056 | *MATα hhf1-hht1::HPH rad30::KAN smc1-259 ade3::GALHO leu2-3,112::PGAL-SMC1-MYC-LEU2 his3-11,15::HIS3-tetR-GFP ura3-1::tetOx112-URA3 ade2-1 trp1-1 can1-100 GAL psi+ RAD5* |
| 1057 | *MATα hht2-hhf2::HPH rad30::KAN smc1-259 ade3::GALHO leu2-3,112::PGAL-SMC1-MYC-LEU2 his3-11,15::HIS3-tetR-GFP ura3-1::tetOx112-URA3 ade2-1 trp1-1 can1-100 GAL psi+ RAD5* |
| 1090 | *MATa set2::HPH smc1-259 ade3::GALHO leu2-3,112::PGAL-SMC1-MYC-LEU2 his3-11,15::HIS3-tetR-GFP ura3-1::tetOx112-URA3 ade2-1 trp1-1 can1-100 GAL psi+ RAD5* |
| 1094 | *MATa set2::HPH rad30::KAN smc1-259 ade3::GALHO leu2-3,112::PGAL-SMC1-MYC-LEU2 his3-11,15::HIS3-tetR-GFP ura3-1::tetOx112-URA3 ade2-1 trp1-1 can1-100 GAL psi+ RAD5* |
| 1101 | *MATa smc1-259 rad30::KAN ade3::GALHO leu2-3,112::PGAL-SMC1-MYC-LEU2 his3-11,15::HIS3-tetR-GFP ura3-1::tetOx112-URA3 ade2-1 trp1-1 can1-100 GAL psi+ RAD5* |
| 1234 | *MATa smc1-259 leu2-3,112::PGAL-SMC1-MYC-LEU2 his3-11,15::HIS3-tetR-GFP ura3-1::tetOx112-URA3 tor1-1 fpr1::NAT trp1-1::RPL13A-2xFKBP12-TRP1 RPB1-FRB::KAN ade2-1 can1-100 GAL psi+ RAD5* |
