# Supplementary material for: Deficiency of Polη in Saccharomyces cerevisiae reveals the impact of transcription on damage-induced cohesion
Source: PLoS Genet. 2021 Sep 9;17(9):e1009763. doi: 10.1371/journal.pgen.1009763 (PMC8454932; doi:10.1371/journal.pgen.1009763)
Supplement: S2 Table — (DOCX) [file pgen.1009763.s015.docx]

| **S2 Table. Information on used primary antibodies** | |  |
| --- | --- | --- |
| **Antibody** | **Company** | **Catalog #** |
| anti-Rpb1 (8WG16) | Santa Cruz Biotechnology | sc-56767 |
| anti-Cdc11 (y-415) | Santa Cruz Biotechnology | sc-7170 |
| anti-Rad53 | Abcam | ab104232 |
| anti-c-myc | Roche | 11667203001 |
| anti-Htz1 | Active Motif | 39647 |
| anti-Histone H2A (phospho S129) | Abcam | ab15083 |
