# Supplementary material for: Deficiency of Polη in Saccharomyces cerevisiae reveals the impact of transcription on damage-induced cohesion
Source: PLoS Genet. 2021 Sep 9;17(9):e1009763. doi: 10.1371/journal.pgen.1009763 (PMC8454932; doi:10.1371/journal.pgen.1009763)
Supplement: S3 Table — (DOCX) [file pgen.1009763.s016.docx]

| **S3 Table. Primers used in ChIP-qPCR** | |
| --- | --- |
| **Name** | **Sequence** |
| *ECM29* pro F | TACCAGTTTAGCCGCCAG |
| *ECM29* pro R | CGCAATTCTGCCTTCTCC |
| *ECM29* mid F | ATCGCAGACTCGACTCAC |
| *ECM29* mid R | GTCACTTGGCAGACCAAC |
| *ECM29* end F | ACACATGGAGAACGCAAC |
| *ECM29* end R | CTTCTGAATGATCAGGCCAC |
| *RIM4* pro F | CTCTCTTCCTTTCTCTCTCCC |
| *RIM4* pro R | GAGTCGGCCTTTAGACCATTAG |
| *RIM4* mid F | TGGATCATCGAATGGGCAC |
| *RIM4* mid R | CCTCTGAATCACTACCATGCAC |
| *RIM4* end F | TTATCCCATGTCACCACCTCC |
| *RIM4* end R | GGTACTGCCATGATTAGCAGC |
| *MSC1* pro F | GAGAGGGAGGAAACAAGGAG |
| *MSC1* pro R | CGGAAACCGCATTAACCAAC |
| *MSC1* mid F | CAGAAAAGGCAGAACAGCAG |
| *MSC1* mid R | ACCTTGTGGCTCTCCAAC |
| *MSC1* end F | GGTTGTTCGGCACTGTTAAG |
| *MSC1* end R | GTACATTACGTTGACACCCC |
| *NPL4* pro F | GCCCTCGTAACATACAGAAC |
| *NPL4* pro R | GGTCCAGATTACCCACCAAC |
| *NPL4* mid F | GATATACGAGCCCCCTCAG |
| *NPL4* mid R | CAAAAAACAGACCCATCCCC |
| *NPL4* end F | GCAGATACTCTCTCCAGACG |
| *NPL4* end R | TCTCTCCTAGCCGCTTTC |
| lowbinding *TAX4* up F (n1) | CCGAAACTGCAAATCCTCC |
| lowbinding *TAX4* up R (n1) | TTCATCGCTCCTTTCCCC |
| lowbinding *ADH3* up F (n2) | CACATCCCTTTGAAACGCAC |
| lowbinding *ADH3* up R (n2) | TACCCTCGACAAATGCCC |
| 31W *SPF1* pro F | GCTTCTTGTCACGCCATAC |
| 31W *SPF1* pro R | CCCTAACAATAGGACTGCTCAC |
| 31W *SPF1* end F | ACCTGAGCTAAACGAAGCC |
| 31W *SPF1* end R | GCAATCTTGACCTGTTGCAC |
| 037C *RAD23* pro F | GCTAGGCAAGAAATAGCGAC |
| 037C *RAD23* pro R | TCTGCGAACGGCCTTATC |
| 037C *RAD23* end F | GGACAAGGTGAAGGTGAAGG |
| 037C *RAD23* end R | CGGCATGATCGCTGAATAG |
| 039C *CYC7* pro F | GCAAGGGGCAAAGACAAAG |
| 039C *CYC7* pro R | GTATGACACTGCTGACACC |
| 039C *CYC7* end F | GTACCAAGATGGCGTTTGC |
| 039C *CYC7* end R | TCTCCTCCGACGACATAGC |
| 056W *HAT2* pro F | CTTCTGCCTCTCTTATCTCTCC |
| 056W *HAT2* pro R | CGTGTCTCGCTAACAAAGTC |
| 056W *HAT2* end F | GAACAAACACCTGATGACGC |
| 056W *HAT2* end R | CGCCTTTTCGCCAAAGAAAC |
| 060C *PRB1* pro F | CAAACACACCCGCGATAAAG |
| 060C *PRB1* pro R | GGATGACCAAAGCAGCAG |
| 060C *PRB1* end F | ACAACGGTGGTGGTCAAG |
| 060C *PRB1* end R | GGACAAACGATAGTGAAGAGGG |
| Htz1 LBD2 F (n) | TGAGCCAGCCAACTCAGAC |
| Htz1 LBD2 R (n) | AAAAACTACGCCTCCACCC |
