# Supplementary material for: Deficiency of Polη in Saccharomyces cerevisiae reveals the impact of transcription on damage-induced cohesion
Source: PLoS Genet. 2021 Sep 9;17(9):e1009763. doi: 10.1371/journal.pgen.1009763 (PMC8454932; doi:10.1371/journal.pgen.1009763)
Supplement: S4 Table — (DOCX) [file pgen.1009763.s017.docx]

| **S4 Table. Primers used in RT-qPCR** | |
| --- | --- |
| **Name** | **Sequence** |
| *RIM4* end F | TTATCCCATGTCACCACCTCC |
| *RIM4* end R | GGTACTGCCATGATTAGCAGC |
| *SOR1*-F | CGTGACGCTGTGAACTTG |
| *SOR1*-R | GTCTTGACTACCTCTCCACC |
| *DDR48*-F | GCTCCAGCAACAACAATGAC |
| *DDR48*-R | CGACCGCCTCTATTAGATGAAC |
| *PUT1*-F | TCACTTGGTTGTCGCCTC |
| *PUT1*-R | CACAGCATCCCCGTTTTC |
| *ECM29* end F | ACACATGGAGAACGCAAC |
| *ECM29* end R | CTTCTGAATGATCAGGCCAC |
| *NPL4* end F | GCAGATACTCTCTCCAGACG |
| *NPL4* end R | TCTCTCCTAGCCGCTTTC |
| *AAC1*-F | ATGATGACTTCGGGCCAGAC |
| *AAC1*-R | TCGTACAATGAGATGACACCAG |
| *BIO2*-F | TGCCAAAGGCCATTATAAGAC |
| *BIO2*-R | GCAATCCCCATTTAGCCAAC |
| *TPO3*-F | CCTTCCCCTTGTTTACCATTC |
| *TPO3*-R | ACTTATGTCTCAAACCTTTGCC |
| *UBI4*-F | TTCCTCCAGACCAGCAAAG |
| *UBI4*-R | TCAGTTACCACCCCTCAACC |
| *ASI3*-F | TCTCTCCTGCCTAATCTGC |
| *ASI3*-R | ACCCTTCACCTCACTATCAC |
| *ECO1-F* | TTTATGGGGGCAATGGTAAA |
| *ECO1-R* | GTCCTGCACACCCAAATTCT |
| *FBA1*-F | TTCCACGGTGGTTCCGGTTC |
| *FBA1*-R | CTGGGTTACCGACTGGGGAC |
| *luciferase*-2005-F | TACAACACCCCAACATCTTCGA |
| *luciferase*-2005-R | GGAAGTTCACCGGCGTCAT |
