# Supplementary material for: Deficiency of Polη in Saccharomyces cerevisiae reveals the impact of transcription on damage-induced cohesion
Source: PLoS Genet. 2021 Sep 9;17(9):e1009763. doi: 10.1371/journal.pgen.1009763 (PMC8454932; doi:10.1371/journal.pgen.1009763)
Supplement: Attachment — Submitted filename: 210707 Wu et al Rebuttal.docx [file pgen.1009763.s018.docx]

*Response to comments from Editors and reviewers on:*

*“The assisting role of Polη in transcription facilitates formation of damage-induced cohesion” by Pei-Shang Wu et al., PGENETICS-D-21-00364*

*We would like to start by thanking the editors and reviewers for their time spent, as well as their appreciated and insightful comments on our manuscript. Despite the criticism raised, we were encouraged by the interest shown and the comments that were positive. We have now revised our manuscript, starting with a new title; ‘Transcription deregulation driven by absence of Polη brings negative impact on damage-induced cohesion’, which better describes our findings. Below follows a detailed description of experiments and modifications done to the manuscript, in response to the concerns raised by the reviewers.*

Editor comment:

Reviewers 1 and 3 raise a number of major concerns that would need to be addressed experimentally.

*In connection to the comments from reviewers 1 and 3, we describe the new experiments we have performed to try to meet their concerns.*

Reviewer #1: Cohesin binds around DSBs and is important for repair. Interestingly, DSBs trigger a genome-wide reinforcement of cohesion. This manuscript seeks to build on previous work implicating RAD30 in the genome-wide process but not DSB-local cohesion. They use an engineered budding yeast strain with a ts mutation in smc1, engineered to make a gal inducible HO break on chromosome 3 and monitor cohesion on chromosome 5. In a few cases gamma irradiation is used instead of an HO break. The authors investigate how RAD30 contributes to transcription as a mechanism by which it generates genome-wide cohesion. *The hypothesis put forward is that RAD30 facilitates DI-cohesion via its role in the transcriptional response to DSBs*. *The data in support of this hypothesis needs to be stronger* to warrant publication.

*We are happy that the description of our experimental system has been fully comprehended. However we would like to clarify that we do not suggest that the role for Polη in transcription is important exclusively during the transcriptional response to DSBs, rather its role in transcription in general (lines 316-319 in the first submission). Our new experiments, described in Fig 3, further suggest that transcriptional deregulation arises from absence of Polη during multiple cell cycles prior to DSB induction.*

Summary of Figures: In figure 1 they demonstrate Rad30 mutant strains are sensitive to transcriptional inhibitors and Rpb1 ChIP shows it has reduced localization to several genes despite normal protein levels. In figure 2 they demonstrate that gene expression is disrupted in the RAD30 deletion strain under normal growth conditions and the transcriptional response to a DSB is attenuated. The authors argue that this attenuated transcriptional program could underlie the lack of genome-wide damage-induced cohesion. *However, by this logic, any mutation that reduces the transcriptional response to a DSB would have this phenotype-this is a point that should be further probed experimentally.*

*As we reasoned that general transcriptional deregulation would affect formation of damage-induced cohesion, we tested the hir1Δ and htz1Δ mutants that perturb transcriptional activation; as a way to mimic rad30Δ cells. Deletion of HTZ1 for instance, is shown to cause transcriptional misregulation [**1**].*

*To address our question in a more direct way, we tried to inhibit transcription by addition of the transcription inhibitor thiolutin. Thiolutin however in itself provokes a DNA damage response in form of H2AS129 phosphorylation and was therefore not applicable (S4A Fig in the first submission). We have now addressed this question by anchoring Rpb1, the largest subunit of RNA polymerase II, away from nucleus. Please find further information on this, described under ‘major concerns-point 2’ and in the revised manuscript (Fig 7F).*

In Figure 3, they show that rad30 mutants have more cohesin specifically at the TSS, with no difference in the TES, and irrespective of DSB induction. The relationship of this information to the main point could be more clearly stated.

*We have now moved and modified the text describing these results to Figs 4 and S4, hopefully clarifying why they are relevant.*

Figure 4 shows the gene expression signatures, further analyzing the data from Figure 2. In the next section of the manuscript the authors switch to genetic approaches to probe chromatin proteins that influence DI-cohesion, based in part on the gene expression data in the rad30 mutant. In figure 5 they show that *deletion of HIR1 partially rescues DI cohesion in the Rad30 mutant background* whereas histone deletions have no DI-cohesion defects and do not rescue.

*We do not actually believe that the HIR1 deletion rescues the Polη deficiency. We make this conclusion from comparing the sister separation levels between the hir1Δrad30Δ and rad30Δ mutants during the course of experiments. Rather these results show to us that deleting HIR1 in itself leads to partially deficient damage-induced cohesion, as the sister separation level of the hir1Δ single mutant was in between the WT and rad30Δ cells. This indicates that histone exchange is important for damage-induced cohesion, which was further supported by the defect that we observed in the htz1Δ mutant.*

In figure 6 they test the effect of deleting the Htz1 histone variant. They cannot use the HO system so they induce breaks with radiation. They show that Htz1 levels are higher by ChIP following a break in the rad30 mutant. In Figure 7 they show Set2 can partially rescue DI-cohesion in the rad30 background. They also show ChIP data for Rpb1 but I can’t figure out what I am supposed to take away from the ChIP experiment.

In Figure S7 they attempt to integrate their findings into a working model. I appreciate the effort to show a working model but it needs more work. In summary it seems that Hir1 and Htz1 affect histone turnover and DI-cohesion is defective. *Do they mean to imply that Rad30 affects histone turnover directly? Indirectly?*

*Yes, by observing increased Htz1 occupancy at selected promoters in rad30Δ cells (Fig 6), the data suggest that absence of Polη affects histone turnover at promoters. However, we consider the effect on histone turnover to be indirect as Polη appears to play an indirect role in transcription. This is further addressed by temporal depletion of Polη during G_2_/M phase (revised Fig 3E-F).*

*Why does altered elongation with set 2 deletion rescue the rad30 phenotype?* If DI-cohesion is defective in rad30 due to an attenuated transcriptional response to a DSB, does set2 deletion rescue by rescuing the transcriptional response in rad30? For me, the major question presented in the introduction, which is *whether transcription contributes to genome-wide DI-cohesion*, is an interesting one. But *the manuscript does not meet the bar to answer this question, or even partially answer it with respect to RAD30 function*. Results are not sufficiently integrated with each other, making it *difficult to understand how each experiment addresses the overarching question of how transcription contributes to genome-wide DI cohesion*. The data provided is not sufficient to support the hypothesis that Rad30 deletion blocks the formation of damage induced cohesion via altering the transcriptional program.

*We would like to explain our concept as follows. The question ‘whether transcription contributes to genome-wide damage-induced cohesion’ was raised from our attempt to understand the deficiency of rad30Δ cells in damage-induced cohesion. As information about the potential role of Polη in transcription was limited, we first addressed the transcriptional response of rad30Δ cells before and after DSB induction. After confirming that transcription is dysregulated in rad30Δ cells, we switched to investigating the possible linkage between transcription and damage-induced cohesion by testing mutants that would either mimic or rescue rad30Δ cells, based on their reported phenotypes. This included hir1Δ and htz1Δ mutants that would mimic rad30Δ, as well as the set2Δ mutant that would rescue rad30Δ cells from transcriptional defect. The idea that ‘transcription contributes to genome-wide damage-induced cohesion’ is now strengthened by demonstrating that transcription inhibition hampers formation of damage-induced cohesion in Fig 7F.*

*The question concerning the set2Δ mutant is further answered under the ‘major concerns’, point 3.*

Furthermore, *its not clear whether the effect is direct or indirect*. I have suggested three experiments below that would potentially begin to further examine the hypothesis, but depending on the outcome, more experiments may be warranted. This includes *ChIP of Rad30, to be integrated with gene expression data, inhibiting transcription and examining DI-cohesion, and further experimentation to understand how deletion of Set2 rescues Rad30.*

**Major concerns and suggestions for how to improve the manuscript**

*We thank referee #1 for insightful suggestions for improvement of the manuscript. Here we describe in detail how we have tackled these concerns point by point.*

1. Are the promoter types that are associated with differentially expressed genes in the rad30 mutant promoters at which Rad30 binds? This information would help argue for a direct role for Rad30 in the transcription of these genes, and would help support the model.

*To address this question, we performed a Polη-ChIP-sequencing analysis. In addition to our previous Polη-ChIP-on-chip map from S-phase cells [**2**], there is to our knowledge only one report showing chromatin binding of Polη [**3**]. Since Gali et al used a MYC tag for the ChIP and saw a significant signal over an untagged control, we decided to try a MYC tag on RAD30 instead of the FLAG tag that we previously utilized. After running a pilot Polη-ChIP-qPCR, we were encouraged to further analyze genome-wide distribution of Polη by ChIP-sequencing. The results showed that Polη preferentially associated with closed and TATA-containing promoters (Fig 3), and nicely correlated with the prediction that we obtained from the rad30Δ RNA-seq results (Table 1).*

2. It’s a shame thiolutin induced a DNA damage response on its own and prevented them from testing DI-cohesion when transcription was inhibited, as this could help support the model. Could the authors find a different way to do this experiment? What about ActD treatment?

*As an alternative to thiolutin, we tested both actinomycin D and alpha amanitin for inhibition of transcription. Considering the low permeability of yeast cells to these drugs, we futher applied polygodial to improve cells’ permeability [**4**]. However, we found that these treatments were not inhibiting transcription efficiently during a time period plausible for our damage-induced cohesion assay. We therefore created a yeast strain where Rpb1, the largest subunit of RNA polymerase II, can be anchored away from the nucleus by addition of rapamycin. As shown in S7D Fig, Rpb1 was evenly distributed over the cytoplasm after 1 hour incubation with rapamycin. This resulted in approximately two-fold inhibition in expression of selected genes (S7E Fig), without provoking early DNA damage response (S7F Fig). By addition of rapamycin or not, we showed that inhibition of transcription affects DI-cohesion negatively (Fig 7F). In these experiments we used γ-irradiation as the source of DSBs, since we wanted to induce expression of the ectopic P_GAL_-SMC1-MYC prior to transcription inhibition, as well as inhibiting transcription before DSB induction. The P_GAL_-HO allele was therefore not applied in these experiments. In addition, the slightly higher separation of sisters in irradiated WT cells in Fig 7F, compared to WT cells in Figs 6A and S6B, could depend on the shortened recovery period after γ-irradiation. This experimental design was used to avoid cells escaping from the G_2_/M arrest, because of the prolonged experiment with the additional one-hour rapamycin treatment. This might have led to fewer cells in the population forming damage-induced cohesion, compared to those in Figs 6A and S6B.*

3. It’s not clear to me *why the authors decided to examine the specific chromatin mutants chosen (Hir1, Htz1, Set2)*. *Were these genes highly dysregulated in the gene expression data or chosen some other way?* To me the manuscript seems to jumps around from one mutant to another without much logic and just when we start to understand how one mutation is acting they move to another. A more in depth analysis would be helpful to arrive as a more mechanistic understanding, perhaps focusing on *how deletion of set2 rescues rad30*. One experiment that could be done is examine whether the double mutant has a transcriptional profile that is more wild-type relative to the rad30 mutant profile, which would support the model.

*These mutants were selected as they would either mimic or rescue rad30Δ cells in transcriptional deregulation, a concept we also try to explain above. Perturbing histone exchange at promoters by deletion of HIR1 or HTZ1 is correlated with transcriptional activation. In addition, genome-wide transcription is misregulated in the htz1Δ mutant. Deletion of HIR1 would also affect gene expression besides of the HIR-dependent histone genes, since it was reported that some promoter regions become nucleosome-free upon removal of Hir1 [**5**]. The set2Δ mutant was tested as it appeared to suppress sensitivity of some transcription elongation mutants to transcription elongation inhibitors. The other reason why we tested this mutant was that the histones within coding regions of set2Δ cells become hyperacetylated [**6**], and we thought this would create a chromatin status that mimic cells undergoing active transcription. Furthermore, selection of these mutants is related to the finding that genes involved in chromatin assembly and positive transcription regulation were down regulated in rad30Δ cells (Fig 4A). Besides of the mutants mentioned above, the histone gene-pair mutants (hhf1-hht1Δ and hht2-hhf2Δ) were included (Fig 5B-C) to examine if the role of the HIR complex in histone gene repression is related to formation of damage-induced cohesion, or not. As we were applying a genetic approach to address our question of interest, the switching between different mutants was unfortunately unavoidable.*

*We also tried to address the question about how the SET2 deletion rescues the rad30Δ mutant. By examining cell viabilities on plates containing transcription elongation inhibitors, deletion of SET2 compensated for the absence of Polη during transcription elongation. Based on this, we tested and found that the SET2 deletion similarly rescued rad30Δ cells from deficient damage-induced cohesion. These results together implied that the deficiency of rad30Δ cells is relevant to its transcription deregulation, and we furthermore showed that deleting SET2 subtly compensated for the reduced Rpb1 chromatin binding in rad30Δ cells during G_2_/M phase. We also tested, but do not show that the SET2 deletion did not improve gene expression levels in rad30Δ cells. This could be due to the limited number of genes tested by RT-qPCR. However, in a similar fashion, deletion of SET2 did not affect gene expression but caused increased Rpb1 binding at 3’-end of genes as reported by Kelby O. Kizer et. al (their Fig 5D) [**7**]. The set2Δ mutant therefore provided a genetic implication in our study, while we regrettably can only present limited information about the underlying mechanism for how the SET2 deletion rescues the rad30Δ cells.*

Minor concerns
4. Please add relevant statistics to Figure 2 D and E to demonstrate the change from WT.

*Statistics has been added.*

5. I’m not sure the results regarding short genes in figure 2F are relevant to the overall message.

*These results are included as an additional indication of transcriptional deregulation in rad30Δ cells. We have slightly modified the description of these results and hopefully it is now more reasonable that they are presented.*

6. The cohesion defect for RAD30 in part 5A is much larger than in part C and so a partial rescue would be much harder to observe.

*We are aware of the fact that the level of sister separation differs between experiments. One reason for the variation in separation of sisters, at least for WT cells, could be that the population of Polη that is activated and can participate in formation of damage-induced cohesion varies between experiments. We therefore always include WT and rad30Δ cells as controls. To all samples that we assessed for sister separation, DAPI was added for visual confirmation of proper G_2_/M arrest of the cells counted. In this way we always compared the relative separation of sisters between WT, rad30Δ and mutants in the same set of biological repeats, thereby determining the ability of a mutant to be proficient or deficient in damage-induced cohesion. Based on this, we interpreted the levels of sister separation between rad30Δ and hir1Δrad30Δ mutants as similar, albeit the latter showed slower sister separation. The levels of sister separation were comparable between the rad30Δ and the hhf1-hht1Δrad30Δ, or hht2-hhf2Δrad30Δ cells.*

7. For Figure 6, the authors should check Htz1 proteins levels by western to help with interpretation of the ChIP. Are the levels staying the same or going up in the rad30 mutant?

*Thank you for pointing this out, this has now been done. As can be seen in the new Fig 6D, the protein level of Htz1 comparing WT and rad30Δ cells are near-indistinguishable. This indicates that absence of Polη affects the exchange of Htz1 for H2A at promoters. This might be in line with the reduced RNA polymerase II at promoters in rad30Δ cells, as reversely, blocking formation of the transcription preinitiation complex caused retention of Htz1 at promoters [**8**].*

8. The authors have used a graphical image of budding yeast cartoon to diagram cohesion but it doesn’t print well in my copy. It needs a black outline.

*We have modified the cartoon and hope that it is more visible and printable in the new version.*

Reviewer #2: In their manuscript PGENETICS-D-20-00364 Wu and colleagues attempts to define the contribution of the yeast TLS DNA Pol eta to DNA damage-induced cohesion formation, a phenomenon the same group reported in 2013 in Plos Genetics. Here, they conclude that Pol eta affects damage-induced cohesion through its novel role in transcription. First they confirm the role of Pol eta in transcription, previously reported by others, using RNA seq., establishing that a few hundred genes are up or downregulated in the absence of the RAD30 gene coding for Pol eta. The reduced association of RNA PolII at promoters and coding regions in rad30 delta detected by ChIP-qPCR agrees with a transcriptional regulatory role of Pol eta. Intriguingly, cohesin association at promoters is increased in rad30 delta, still damaged-induced cohesion is impaired. They found similar deficiency when deleting HIR1 needed for H3 exchange for transcription activation, and suppression of the rad30 delta transcriptional and cohesin formation defects by deletion of the SET2 histone methyl transferase repressing histone exchange during transcription elongation. Based on these they conclude that deregulated transcription in rad30 delta, which perturbs dynamic nucleosome assembly affects formation of damage-induced cohesion. The topic of the paper DNA damage induced cohesion formation is highly interesting, and the results represent substantial contribution. The experiments are carefully executed with all the necessary controls, and the paper is clearly written. I have one concern regarding the experimental repetitions. The authors state that the results indicate the mean ± STDEV of at least two independent experiments, in almost all figure legends. Two experiments are not enough for solid statistics and conclusions, especially when STDEV is high. *They should indicate the exact numbers of biological and technical replicates for each experiment, and perform additional repetitions if necessary.*

*We thank you for the encouraging words and hope that we in this revised manuscript have been able to respond to the issues raised. For each figure legend, we have now indicated the number of biological repeats performed. We consider statistics on two experiments to be possible if each data point includes multiple events, and that the population in question is expected to display a normal standard distribution. This applies to the damage-induced cohesion experiments where we counted ≥200 cells for each time point, for each individual experiment. Assays where high variation between experiments was expected, or appeared to be the case, such as RNA-sequencing and some of the ChIP-qPCR analyses, were performed in triplicates. In addition, most of the experiments performed during revision were done in triplicates, as advised.*

Reviewer #3: This manuscript investigates the potential links between transcription and damage-induced cohesion through a common role of Rad30, the DNA polymerase eta involved in translesion synthesis. The authors first tested the toxicity of transcription inhibitors in rad30D strains and observed increased sensitivity as well as a decrease in the amount chromatin bound PolII. Similar observations were obtained with the DI-cohesion mutant of Rad30, Rad30-S14A. The authors observed deregulation of transcription profiles for Rad30D in the presence and absence of DNA damage hence suggesting a role for Rad30D in general transcription regulation. Moreover, *the levels of cohesin binding at TSS in Rad30D cells were increased and this led the authors investigate the link between DI-cohesion and transcription at promoter sites.* To this aim they used hir1D and htz1D mutations, which disrupt histone assembly at gene promoters. They found that both mutants decrease the ability of cells to establish DI-cohesion, thus concluding that histone exchange disruption at promoters causes defects in damage-induced cohesion. Finally, the authors show that deletion of histone methyltransferase set2 suppresses the DI-cohesion defects of Rad30. The authors conclude from this data that transcription influences formation of damage-induced cohesion, and that Rad30 is required for damage-induced cohesion because it facilitates transcription.

*We would like to here explain the reason why we further looked into cohesin binding in rad30Δ cells. We realized that the reasoning for this analysis was unclear, and this may have raised some confusion. We performed additional analyzis of cohesin binding at TSS and TES in WT and rad30Δ cells, after knowing that transcription is deregulated, and the global nucleosome occupancy is moderately increased in the rad30Δ mutant. It was also based on the knowledge that the transcription machinery has been proposed to move cohesin from its loading sites to the intergenic region of convergent genes, and that cohesin loading requires nucleosome-free regions. Therefore, it was possible that cohesin loading would be reduced specifically at TSS in rad30Δ cells. Based on the additional metagenome analyses performed here, this possibility was however excluded. The reason why we tested the hir1Δ and htz1Δ mutants for formation of damage-induced cohesion, was mainly because perturbing histone exchange will affect transcriptional activation, and we considered that this would mimic the situation in rad30Δ cells.*

Overall, this manuscript makes some interesting observations and indeed makes some weak links between Rad30, transcription and DI-cohesion. It has been previously shown that cohesin loading requires nucleosome free regions and *therefore changes in transcription or nucleosome assembly/chromatin at promoters (in hir1D and hzt1D) are indeed expected to have an impact on DI-cohesion.* The role of Rad30 is however intriguing and although the changes in transcription in Rad30D cells could indeed explain some effects, the links are very vague and often do not offer a clear cut explanation of how Rad30 might be either involved in transcription regulation or facilitate DI-cohesion (by altering transcription), as the authors only offer some genetic interactions. Also, the use of Rad30D mutants might not be the best experimental system.

*We would like to clarify that perturbing histone exchange by deletion of HIR1 or HTZ1, should not affect formation of nucleosome-free region. The histone exchanges take place at the -1 and +1 nucleosomes, which are the boundary of nucleosome-free regions. It is not eviction of the histone octamers, but histone exchange that happens between H3 and posttranslationally modified H3 (in budding yeast), or between Htz1 and H2A within the nucleosome. It was also reported that some promoters even become nucleosome-free when HIR1 is deleted [**5**]. In addition, concerning cohesin loading in these mutants, the hir1Δ and htz1Δ mutants showed no defect in maintenance of sister chromatid cohesion that was formed during replication, as reported by Jingjing Zhang et. al [**9**] for the former, and in our revised S6C Fig for the latter.*

The authors should use Rad30 auxin degron strains where they could remove Rad30 upon DNA damage and avoid the background effect of the deletion.
To support their claims it would be important for the authors to illustrate their points using a handful of specific genome sites *where they can investigate changes in transcription and cohesin loading (or cohesion establishment) upon damage in a manner dependent on Rad30 (using an auxin degron approach)*. This would significantly strengthen their study.

*We have previously made attempts to perform damage-induced cohesion experiments in a Polη-AID strain combined with the Tet-off system for efficient reduction of the Polη level. However, we found that depletion of Polη was inefficient at 1 mM auxin and 20 μg/ml doxycycline (final concentrations). We could potentially have increased the final concentration of auxin to 6 mM as we did for the later RT-qPCR analyses (Figs S3B, S3D and 3F), but we also found that the LacO/LacI-GFP dots for determination of damage-induced cohesion became extremely weak under these conditions, and the results would not be trustworthy. We therefore did not further try to perform damage-induced cohesion experiments with this ‘Polη-AID+Tet-off’ strain.*

*We have here instead used the ‘Polη-AID+Tet-off’ strain to monitor expression of Polη-bound genes, selected based on our Polη-ChIP-seq analysis. We find that these genes are downregulated in rad30Δ cells but not after depletion of Polη specifically during G_2_/M (Fig 3D-F). These experiments indicate that the role for Polη in transcription is indirect and the transcriptional deregulation that we observed in rad30Δ cells is built up during several cell cycles.*

*We previously concluded that cohesin binding or its de novo recruitment is generally not affected in the rad30Δ cells. Our current metagenome analyses in addition showed that cohesin binding is increased around TSS in the rad30Δ mutant. Considering these findings, it is unlikely that the cohesin binding or the de novo recruitment of cohesin is affected by temporal depletion of Polη.*

*Lastly, we would again like to thank all the reviewers and editors for your time and suggestions on our work. We hope that your comments have been answered. We look forward to hearing from you about our manuscript after revision, and we will be happy to respond to any additional questions or comments that you may have.*

Related references

1. Morillo-Huesca M, Clemente-Ruiz M, Andujar E, Prado F (2010) The SWR1 histone replacement complex causes genetic instability and genome-wide transcription misregulation in the absence of H2A.Z. PLoS One 5: e12143.

2. Enervald E, Lindgren E, Katou Y, Shirahige K, Strom L (2013) Importance of Poleta for damage-induced cohesion reveals differential regulation of cohesion establishment at the break site and genome-wide. PLoS Genet 9: e1003158.

3. Gali VK, Balint E, Serbyn N, Frittmann O, Stutz F, et al. (2017) Translesion synthesis DNA polymerase eta exhibits a specific RNA extension activity and a transcription-associated function. Sci Rep 7: 13055.

4. Mayan MD (2010) Drug-induced permeabilization of S. cerevisiae. Curr Protoc Mol Biol Chapter 13: Unit 13 12B.

5. Fillingham J, Kainth P, Lambert JP, van Bakel H, Tsui K, et al. (2009) Two-color cell array screen reveals interdependent roles for histone chaperones and a chromatin boundary regulator in histone gene repression. Mol Cell 35: 340-351.

6. Venkatesh S, Smolle M, Li H, Gogol MM, Saint M, et al. (2012) Set2 methylation of histone H3 lysine 36 suppresses histone exchange on transcribed genes. Nature 489: 452-455.

7. Kizer KO, Phatnani HP, Shibata Y, Hall H, Greenleaf AL, et al. (2005) A novel domain in Set2 mediates RNA polymerase II interaction and couples histone H3 K36 methylation with transcript elongation. Mol Cell Biol 25: 3305-3316.

8. Tramantano M, Sun L, Au C, Labuz D, Liu Z, et al. (2016) Constitutive turnover of histone H2A.Z at yeast promoters requires the preinitiation complex. Elife 5.

9. Zhang J, Shi D, Li X, Ding L, Tang J, et al. (2017) Rtt101-Mms1-Mms22 coordinates replication-coupled sister chromatid cohesion and nucleosome assembly. EMBO Rep 18: 1294-1305.
