# Supplementary material for: Deficiency of Polη in Saccharomyces cerevisiae reveals the impact of transcription on damage-induced cohesion
Source: PLoS Genet. 2021 Sep 9;17(9):e1009763. doi: 10.1371/journal.pgen.1009763 (PMC8454932; doi:10.1371/journal.pgen.1009763)
Supplement: Attachment — Submitted filename: 210803 Wu et al Rebuttal.docx [file pgen.1009763.s019.docx]

**Comments to the Authors:
Please note here if the review is uploaded as an attachment.**

Reviewer #1: I appreciate that the authors executed and added a number of additional experiments to address reviewers’ concerns. The overarching question is quite interesting. Most of the individual experiments have proper controls, repeats, and quantification. However, I do not think the authors have reached the bar. The third reviewer commented in the first round of reviews that the link between Rad30, transcription, chromatin, and DI-cohesion is vague, and I think this problem persists in the revised version, although it has improved some in terms of knitting together a story. The constitutive genetic deletion approach makes it difficult to ascertain how the mutations are affecting transcription in the short term and long term, and if the effects are direct versus indirect, and how this corresponds with the effect on the loss of damage induced cohesion. All the null backgrounds allow for transcription to persist, so it must be something more specific about the transcriptional process that leads to loss of damage-induced cohesion. The concerns below are not an exhaustive list of weaknesses. Even if these issues could be addressed, the manuscript may still not meet the bar.

1. New evidence is presented suggesting that the effect of Rad30 deletion is probably indirect as the binding does not correlate with transcriptional changes. This correlation between binding and transcription, or lack thereof, would have been much stronger if the experiments were performed genome-wide and not at a handful of genes.

*In the first revised version of our manuscript we included results from genome-wide Polη ChIP-sequencing, which showed that Polη preferentially binds at closed-, FN- and TATA-containing promoters (Fig 3A-C). This indeed correlated well with the finding that genes being differentially expressed in rad30Δ, compared with WT cells, are regulated mainly by closed and TATA-containg promoters, as implicated by a combination of our RNA-sequencing results and analysis of published databases (Table 1). Hence, we concluded that Polη binding at closed-, FN- and TATA-containing promoters correlates with the altered transcriptional response observed in rad30Δ cells (manuscript R1, lines 169-172).*

*To understand better if the transcriptional deregulation seen in rad30Δ cells was a direct or indirect effect, we compared gene expression in the rad30Δ strain with that of a ‘Polη-degron’ strain, which allowed depletion of Polη temporally during G_2_/M phase (Figs 3E, 3F and S3D). We started with a limited number of genes representing the different types of promoters where Polη was shown to bind or not (Fig 3D). We certainly agree that a genome-wide analysis would provide a more complete picture. However, before proceeding to large-scale investigations, we seek indications of a positive outcome from pilot studies, considering both time and cost. Since there was no direct correlation between binding of Polη at the selected promoters and expression of the corresponding genes after temporal depletion of Polη during G_2_/M, as opposed to the situation in rad30Δ cells, we concluded that a genome-wide gene expression analysis of the ‘Polη-degron’ strain with RNA-sequencing was not motivated.*

2. It’s not clear to me if loss of Rad30 changes histone turnover or histone acetylation. It seems premature to conclude that Rad30 perturbs transcription through a similar mechanism to either Htz1 or Hir1, based on the data included.

*The Htz1 ChIP-qPCR results (Fig 6B and C) showed that there was a significant proportion of Htz1 retained on chromatin, especially after DSB induction, indicating that its exchange for H2A was reduced in the rad30Δ cells.*

*In addition, formation of damage-induced cohesion was negatively affected by deletions of HTZ1 (Fig 6A) and HIR1 (Fig 5A, and related results in Fig 5B-C).*

*From these results, it appeared that histone exchange was negatively affected by the absence of Polη, suggesting a potential correlation between histone exchange and damage-induced cohesion. We did propose this potential connection but also stated that ‘precisely how the persistent absence of Polη indirectly affects transcription remains to be investigated further’ (manuscript R1, lines 418-420).*

*Regarding your concern, we are not sure if you are specifically referring to the sentence ‘Deregulated transcription in rad30Δ cells, which perturbs histone exchange…’ (manuscript R1, line 430). This sentence was written as part of the proposal, which might be misleading. Our intention was to suggest this possibility, but it remains for future investigations to elucidate. We have in the revised version looked through the phrasing and modified accordingly.*

3. I appreciate that the authors added the experiment to induce the removal of Rpb1 to impact transcription. This is potentially a very important experiment. As a control the authors should validate that the addition of rapamycin alone does not impact damage-induced cohesion in a WT background, since rapamycin will impact the transcriptional program even in the absence of the anchors away allele of Rpb1.

*This is indeed true if the Fpr1 and Tor1 proteins are both present. Fpr1 is one of the FK506 binding proteins (FKBPs), which specifically binds to rapamycin. The Fpr1-rapamycin complex inhibits Tor1, the target of rapamycin, thereby affecting transcriptional responses of certain nutrient-sensitive signaling pathways. Therefore, cells harboring the FPR1 and TOR1 genes are sensitive to rapamycin. To eliminate rapamycin toxicity, the FPR1 gene is deleted (fpr1Δ) and the TOR1 gene is mutated (tor1-1). These genetic modifications are standard when applying the anchor-away technique, in addition to the selected anchor (Rpl13A-FKBP12) and the FRB-tagged target [1]. We apologize for not fully describing this genotype. This information is now added to the text in the second revised version, and not only indicated in the list of strain genotypes.*

*Considering that the effect of rapamycin on transcription is based on the interaction between the Fpr1-rapamycin complex and the Tor1 protein, the effect of rapamycin alone on transcription in the strain used for damage-induced cohesion experiments (Fig 7F) should be negligible, since the FPR1 and TOR1 genes were deleted or mutated as described above. In addition, determining damage-induced cohesion in a clean 'WT' background in the presence of rapamycin will not really be possible, since these cells, for the same reason, will be sensitive to rapamycin during the course of this several hours long experiment.*

*We base this reasoning on the following references:*

*Hardwick, J. S. et al, 1999 [2]*

*In this paper, the genome-wide transcriptional response of rapamycin treated cells was elucidated using DNA microarrays, which showed that rapamycin affected certain cellular pathways mediated by the Tor proteins.*

*Heitman, J. et al, 1990 [3]*

*Heitman et al showed that rapamycin treatment induced G_1_ arrest and cell toxicity in budding yeast. The toxicity of rapamycin was shown to depend on the Fpr1, Tor1 and Tor2 proteins. A functional interaction between these proteins was also suggested.*

*Zheng X. F. et al, 1995 [4]*

*Loewith R. et al, 2002 [5]*

*Since the Fpr1-rapamycin complex does not bind to Tor2 in the TORC2 complex, and Tor2 has an essential function that is insensitive to rapamycin, the TOR2 is not mutated in anchor-away strains.*

*In light of all this, we would argue that performing the suggested experiment, ensuring no effect of rapamycin on damage-induced cohesion in WT cells, is not relevant.*

4. Rad30 seems to have its effect on genes with a specific group of promoters. How should I think about the effect on specific promoters relative to damage induced cohesion? Does the actual gene expression program have any bearing on DI-cohesion? The focus on the genes up and down regulated in the null background, which is a long term effect, and the promoter types bound, confuses the issue of the effect on DI-cohesion for me. Stripping away extraneous information may help convey the message better.

*We found that Polη preferentially binds at closed-, FN- and TATA-containing promoters. Logically the genes regulated by these promoters should also dominate the pool of genes affected by deletion of RAD30. This appeared to be the case as presented in Table 1, except the FN promoter (discussed in manuscript R1, lines 404-407), indicating that altered gene expression largely correlates with the types of promoters that Polη associates with, as a long term effect in rad30Δ cells. The genes regulated by a promoter either defined as closed, containing a TATA box, or characterized by both these features together represent approximately 40% of all budding yeast genes. We thereby believe that the assumed collected dysregulation of transcription, rather than the actual gene expression program, has the potential to be responsible for alterations in chromatin features, resulting in the failure to establish damage-induced cohesion in rad30Δ cells.*

*We would like to restate that the analyses of genome-wide distribution of Polη and preferred promoter types were our attempts to understand the possible role of Polη in transcription. Based on our current data, we were not confident to conclude how these specific promoters relate to damage-induced cohesion and therefore it was not extensively discussed in our manuscript. However, we could further speculate on the topic as follows:*

*In yeast, transcriptional bursting and consequent noisy expression is associated with TATA-containing promoters [6, 7] with ‘closed' chromatin conformations. It has furthermore been suggested that clusters of RNA pol II molecules at 5′ ends of genes, resulting from bursts of initiation are more effective at evicting nucleosomes than single isolated RNA polymerases [8]. The RNA Pol II levels at promoters are decreased in rad30Δ cells (Fig 1B), implying that transcriptional bursting could be reduced in rad30Δ cells. This may be correlated with deregulation of genes governed by the closed and TATA-containing promoters in rad30Δ cells (Table 1). The reduced transcriptional bursting may subsequently affect formation of a certain chromatin state that is required for damage-induced cohesion. Nevertheless, these are still speculative options to explain our results and remain open for investigations.*

*In order to increase visibility of the main messages, we have tried to modify the related text in the manuscript where these matters are discussed.*

Minor concerns

1. The adjusted title is poorly formulated. The authors should state their findings in the positive. A preferable title would be something like “Transcription-related chromatin features facilitate damage-induced cohesion”

*We thank the reviewer for the nice title suggestion. However, although both deregulated transcription in the rad30Δ strain and presumed alterations of chromatin features in the hir1Δ, htz1Δ and set2Δ mutants affect formation of damage-induced cohesion, we have not shown a direct connection between deregulated transcription and altered chromatin features. We can thereby not state a direct cause and consequence relation between these entities in the title. We have reconsidered our previous title to describe our findings in the positive and modified it to ‘Deficiency of Polη in Saccharomyces cerevisiae reveals the impact of transcription on damage-induced cohesion’.*

2. The writing style in the manuscript is improved from the original but it is still often difficult to follow the experiments and the logic.

3. The reply to reviewers has, at some points, a similar problem with clarity.

*We are very sorry that the logic and description of experiments in the manuscript, as well as our answers to reviewers’ comments in the rebuttal, are perceived as difficult to follow and unclear. Without specific examples it is however hard to improve this. We have now gone over the text again and added explanations where needed, as well as tried to simplify and increase clarity.*

4. The quality/size of the figures was so poor in my pdf that I had to download figures one by one, and enlarge them.

*We do apologize for this. Our .tiff files for the individual figures were all of good quality and sharp. The figures have also passed the PACE system of PLOS Genetics. During formation of the joined PDF, the quality deteriorated. For the current revision, we have asked for advice from the editorial board and according to them our figures are fine. The figures were compressed in the PDF to enable complete download of the file from broad internet services. We hope that this explains the quality of our figures in the PDF file.*

5. Given comments 2-4, the reviewer’s work is much more challenging than for an average review assignment. The authors and journal should strive to provide a manuscript in a more easily reviewable state.

*We do of course agree that a reviewer task should not be overwhelming. We are sorry to hear that our manuscript did not fulfill the criteria of being easily reviewable. We hope that we have here been able to answer the remaining concerns, and that the revised manuscript will be found satisfyingly improved.*

Reviewer #2: I accept the modifications.

Reviewer #3: The authors have addressed, or explained, most of my concerns, I find the revised version improved. I am now supportive of publication.

*We thank reviewers #2 and #3 again for their time and valuable comments on our manuscript.*

**References**

1.	Haruki H, Nishikawa J, Laemmli UK. The anchor-away technique: rapid, conditional establishment of yeast mutant phenotypes. Mol Cell. 2008;31(6):925-32. Epub 2008/10/17. doi: 10.1016/j.molcel.2008.07.020. PubMed PMID: 18922474.

2.	Hardwick JS, Kuruvilla FG, Tong JK, Shamji AF, Schreiber SL. Rapamycin-modulated transcription defines the subset of nutrient-sensitive signaling pathways directly controlled by the Tor proteins. Proc Natl Acad Sci U S A. 1999;96(26):14866-70. Epub 1999/12/28. doi: 10.1073/pnas.96.26.14866. PubMed PMID: 10611304; PubMed Central PMCID: PMCPMC24739.

3.	Heitman J, Movva NR, Hall MN. Targets for cell cycle arrest by the immunosuppressant rapamycin in yeast. Science. 1991;253(5022):905-9. Epub 1991/08/23. doi: 10.1126/science.1715094. PubMed PMID: 1715094.

4.	Zheng XF, Florentino D, Chen J, Crabtree GR, Schreiber SL. TOR kinase domains are required for two distinct functions, only one of which is inhibited by rapamycin. Cell. 1995;82(1):121-30. Epub 1995/07/14. doi: 10.1016/0092-8674(95)90058-6. PubMed PMID: 7606777.

5.	Loewith R, Jacinto E, Wullschleger S, Lorberg A, Crespo JL, Bonenfant D, et al. Two TOR complexes, only one of which is rapamycin sensitive, have distinct roles in cell growth control. Mol Cell. 2002;10(3):457-68. Epub 2002/11/01. doi: 10.1016/s1097-2765(02)00636-6. PubMed PMID: 12408816.

6.	Raser JM, O'Shea EK. Control of stochasticity in eukaryotic gene expression. Science. 2004;304(5678):1811-4. Epub 2004/05/29. doi: 10.1126/science.1098641. PubMed PMID: 15166317; PubMed Central PMCID: PMCPMC1410811.

7.	Zenklusen D, Larson DR, Singer RH. Single-RNA counting reveals alternative modes of gene expression in yeast. Nat Struct Mol Biol. 2008;15(12):1263-71. Epub 2008/11/18. doi: 10.1038/nsmb.1514. PubMed PMID: 19011635; PubMed Central PMCID: PMCPMC3154325.

8.	Perales R, Erickson B, Zhang L, Kim H, Valiquett E, Bentley D. Gene promoters dictate histone occupancy within genes. EMBO J. 2013;32(19):2645-56. Epub 2013/09/10. doi: 10.1038/emboj.2013.194. PubMed PMID: 24013117; PubMed Central PMCID: PMCPMC3791371.
